# Supplementary material for: MetaRibo-Seq measures translation in microbiomes
Source: Nat Commun. 2020 Jun 29;11:3268. doi: 10.1038/s41467-020-17081-z (PMC7324362; doi:10.1038/s41467-020-17081-z)
Supplement: Supplementary file 10 — Supplementary Data 7 [file 41467_2020_17081_MOESM10_ESM.zip › File2/Confidence_High_Taxonomy/405141_out.krona.html]

Javascript must be enabled to view this page.

members
magnitude
magnitudeUnassigned
count
unassigned
taxon
rank

405141\_out

3

2
superkingdom
3

1239
phylum
3

3
186801
class

186802
order
3

3
541000
family

3
genus
216851

3
853
species

SRS019161\_contig\_number\_37895SRS062654\_contig\_number\_contig-100\_3503.142456SRS098644\_contig\_number\_44864
